# Strong Structural and Property Anisotropy in CeO_2_‐NiFe Hybrid Metamaterials Toward Self‐Assembled Magnon Nanostructures

**DOI:** 10.1002/smsc.202500070

**Published:** 2025-08-06

**Authors:** Lizabeth Quigley, Juanjuan Lu, Claire A. Mihalko, Jialong Huang, Jeremy Gan, Katrina Evancho, Max Chhabra, Raktim Sarma, Aleem Siddiqui, Ping Lu, Haiyan Wang

**Affiliations:** ^1^ School of Materials Engineering Purdue University West Lafayette IN 47907 USA; ^2^ Sandia National Laboratories Albuquerque NM 87185 USA; ^3^ Center for Integrated Nanotechnologies Sandia National Laboratories Albuquerque NM 87185 USA; ^4^ School of Electrical and Computer Engineering Purdue University West Lafayette IN 47907 USA

**Keywords:** ferromagnetic resonance, magnetic anisotropy, microstructural tuning, Ni_80_Fe_20_, transmission electron microscopy, vertically aligned nanocomposite

## Abstract

Ni_80_Fe_20_ (NiFe) is a well‐studied conducting ferromagnet with spin‐transfer torque and current‐driven domain wall motion demonstrated and thus is considered as a potential candidate for magnonics and energy‐efficient computational devices. Various NiFe‐based nanowire and nanotube structures have been made via lithography patterning methods for magnonics demonstration. In this work, through the concept of self‐assembled vertically aligned nanocomposite (VANs) thin films, vertically aligned NiFe (80:20) nanopillars are uniformly grown into an insulating dielectric oxide matrix (CeO_2_) as a hybrid metamaterial framework for nanoscale magnonics demonstration. The NiFe‐CeO_2_ VAN system is compared with a single layer NiFe film based on film morphologies, magnetic anisotropy, and ferromagnetic resonance (FMR) properties. Because of the unique out‐of‐plane (OOP) alignment of the NiFe nanopillars, strong OOP anisotropy and OOP FMR properties are achieved in the NiFe‐CeO_2_ VAN system. This NiFe‐based hybrid metamaterial framework presents as a viable alternative materials solution toward nanoscale magnon applications.

## Graphical abstract

This work explores the effects of the vertically aligned nanocomposite (VAN) design has on the magnetic properties of NiFe, leading to strong out‐of‐plane magnetic anisotropy that the NiFe single layer does not display. The magnon dynamics of the VAN film is also explored, showing the first ever demonstration of ferromagnetic resonance in a NiFe‐based VAN film.

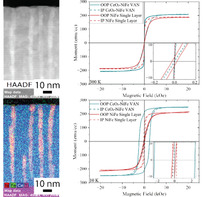

## 1 Introduction

In the past few years, magnetic materials exhibiting magnonic properties have been explored extensively due to their great potential in energy‐efficient memories and data storage devices.^[^ ^1^, ^2^, ^3^, ^4^ ^]^ By definition, magnons themselves are the collective excitations of spin waves within a magnetic material when under an applied magnetic field, typically seen in ferromagnetic and antiferromagnetic materials.^[^ ^5^, ^6^, ^7^, ^8^ ^]^ Understanding how the magnons react under the applied field provides information about the magnetic interactions within the material. One of the key interactions is the magnetic damping, also known as the Gilbert damping coefficient (α), which represents the loss of energy as the spin wave propagates throughout the material.^[^ ^9^, ^10^ ^]^ A lower α indicates a more efficient magnon transport, which is important for spintronic applications.^[^ ^9^, ^10^ ^]^ The magnons can transfer information throughout a material, allowing for low power, high speed, and energy‐efficient devices; however, different magnonic materials are able to achieve magnon transport with different efficiencies and speeds.^[^ ^11^, ^12^, ^13^, ^14^, ^15^, ^16^, ^17^ ^]^ The typical magnonic materials demonstrated so far include Ni_80_Fe_20_ (NiFe in short for the remainder of the paper), Y_3_Fe_5_O_12_ (YIG), CoFeB, and La_0.7_Sr_0.3_MnO_3_ (LSMO) with α values ranging from 1 × 10^−2^ to 1.5 × 10^−3^, 2.2 × 10^−4^ to 6 × 10^−5^, 3.2 × 10^−2^ to 3.61 × 10^−3^, and 2.63 × 10^−3^ to 8.6 × 10^−4^, respectively.^[^ ^11^, ^12^, ^13^, ^14^, ^15^, ^16^, ^17^, ^18^, ^19^, ^20^, ^21^, ^22^ ^]^ Despite the incredibly low α of YIG, it is very challenging to grow YIG in its proper phase on conventional single crystal substrates such as silicon or SrTiO_3_ (STO). Instead, a Gd_3_Ga_5_O_12_ (GGG) substrate is required to stabilize the ferrimagnetic cubic phase of YIG.^[^ ^11^, ^12^ ^]^ Other materials are easier to deposit on conventional substrates and can still achieve low, α including and NiFe.^[^ ^13^, ^14^ ^]^ Because of the relatively easy growth and on‐chip integration, as well as materials compatibility, NiFe still attracts significant attention for magnon transport study and magnon–magnon coupling demonstrations as a conducting ferromagnet.^[^ ^16^, ^23^, ^24^ ^]^

Recently, various NiFe‐based structures for magnon transport study have been made via lithography patterning methods for magnonics demonstration.^[^ ^18^, ^25^, ^26^, ^27^ ^]^ The fabrication of these nanostructures for magnon applications often involves multiple processes, including thin film deposition, lithography, and patterning steps. NiFe films can be deposited using a variety of methods to enhance the transport properties, including patterning the film after deposition and making arrays of nanowires or nanotubes.^[^ ^18^, ^25^, ^26^, ^27^ ^]^ For example, NiFe nanostructures prepared by a lithography method, has been reported to present current‐driven domain wall motion with Gilbert damping coefficient of 1 × 10^−2^ to 1.5 × 10^−3^ and domain wall velocity of 0.5–110 m s^−1^ depending on the ratio of Ni to Fe and film quality.^[^ ^15^, ^16^, ^17^, ^18^, ^28^ ^]^ However, these approaches can often be tedious and challenging due to the multiple lithography and etching steps involved.

Compared to the above‐mentioned conventional lithography and patterning methods, self‐assembled oxide‐metal vertically aligned nanocomposites (VANs) offer the growth of vertical metallic nanopillars uniformly distributed in an oxide dielectric matrix.^[^ ^29^, ^30^, ^31^ ^]^ This self‐assembly VAN growth approach decreases the complexity of the deposition and patterning steps while still allowing for tuning of the nanostructures.^[^ ^29^, ^30^, ^31^, ^32^, ^33^ ^]^ This can be accomplished by varying the deposition parameters, the spacing, density, and diameter of the metallic nanopillars could be adjusted to achieve desired physical properties.^[^ ^29^, ^30^, ^31^, ^34^ ^]^

**Figure 1:**
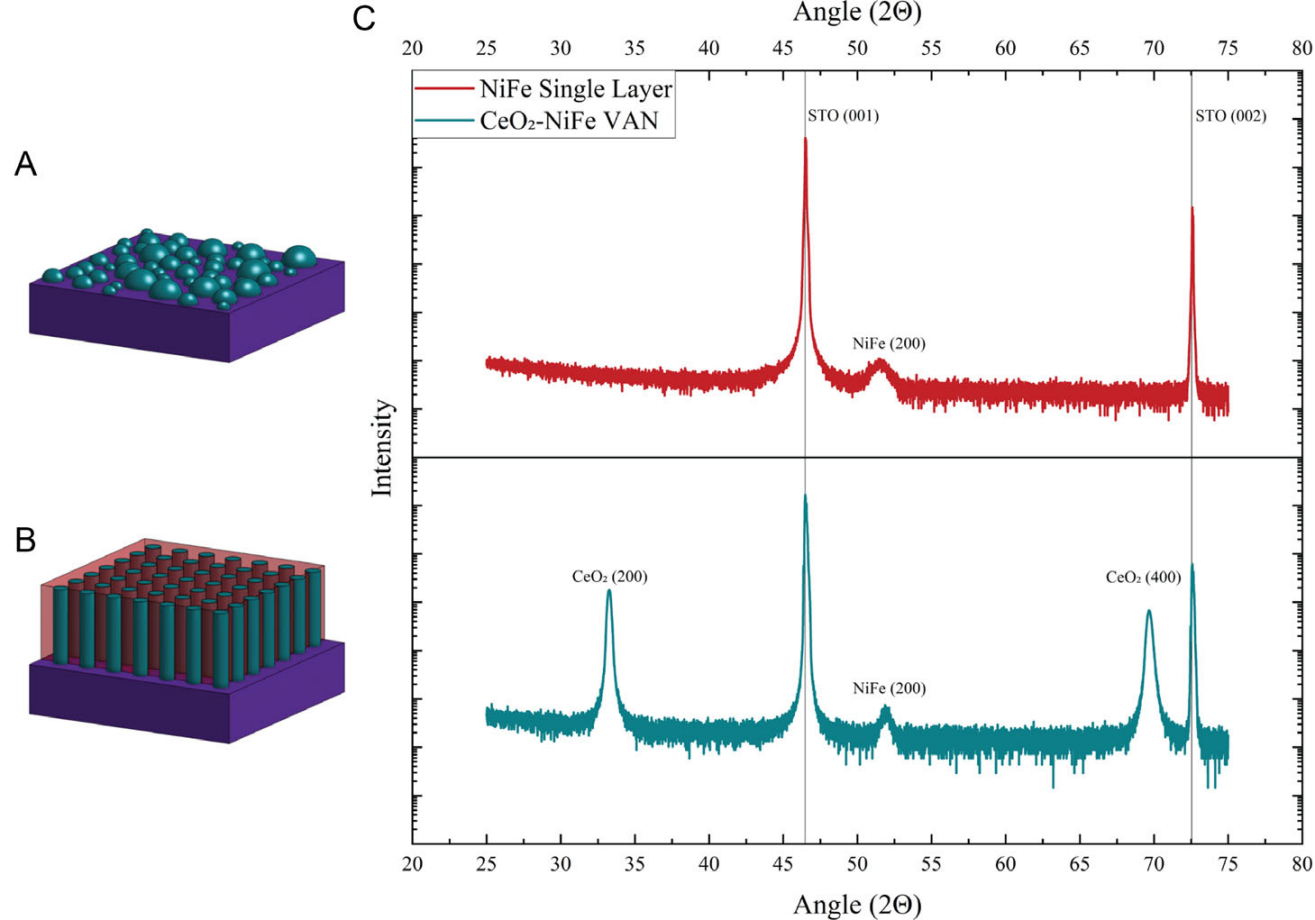
A) Schematic drawing of the as‐grown single‐layer NiFe film, B) schematic drawing of the as‐grown VAN CeO_2_‐NiFe film, and C) XRD *θ*‐2*θ* scan for the NiFe single layer and CeO_2_‐NiFe VAN films.

In this work, a new VAN system of CeO_2_‐NiFe is proposed for this nanostructured NiFe magnon demonstration. CeO_2_ is selected as the insulating dielectric matrix, and the ferromagnetic conducting NiFe as the pillars, in the VAN system as illustrated in **Figure** **1**B. CeO_2_ is diamagnetic, so it would not affect the magnetic properties of the NiFe pillars, allowing for the shape of the pillars to be the main effect compared with the single layer NiFe. CeO_2_ has also been demonstrated as a successful matrix in various metal‐oxide VAN systems with the metallic materials as the pillars, such as CeO_2_‐Au, CeO_2_‐Co, and CeO_2_‐Ni.^[^ ^34^, ^35^, ^36^, ^37^ ^]^ The large lattice mismatch between the two phases in the VAN film and the substrates could allow the formation of a highly strained CeO_2_ layer, as illustrated in Figure 1. The internal strain of CeO_2_ helps grow well defined pillars, with other microstructures not widely reported.^[^ ^34^, ^35^, ^36^, ^37^ ^]^ This work will also deposit a NiFe single‐layer film as a reference (as illustrated in Figure 1) to compare with that of the CeO_2_‐NiFe VAN film, on STO (001), to allow for direct comparison between the single layer and the VAN. All films were fabricated with pulsed laser deposition (PLD), with deposition details in the experimental section. Structural characterization was conducted with X‐ray diffraction (XRD), transmission electron microscopy (TEM), scanning TEM (STEM), and energy‐dispersive X‐ray spectroscopy (EDS). The magnetic characterization included hysteresis loops (M‐H) and ferromagnetic resonance (FMR) to determine how changes in the NiFe microstructure between VAN films and single‐layer films affected the overall magnetic behavior and magnonic transport.

## 2 Results and Discussion

**Figure 2:**
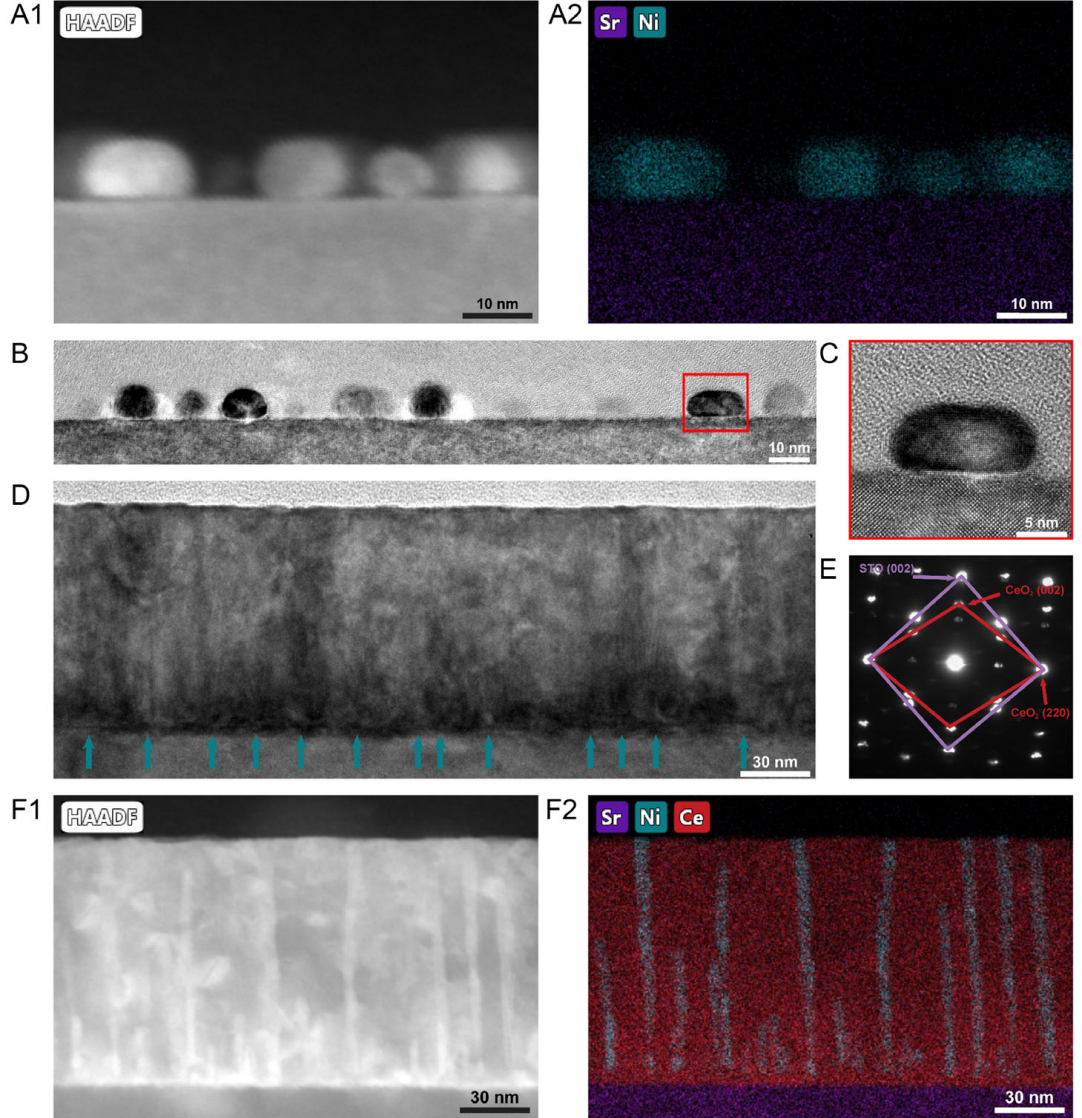
For the NiFe single‐layer sample: A1) STEM image and A2) corresponding EDS maps representing the Sr and Ni element distributions, B) TEM image showing the size and spacing of the NiFe islands, C) enlarged view of one NiFe island. For the CeO_2_‐NiFe VAN sample: D) TEM image of the film with pillars marked with arrows, E) SAED pattern F1) STEM‐HAADF image and F2) corresponding EDS maps representing the Sr, Ni, and Ce elements.

A pure NiFe thin film and a CeO_2_‐NiFe VAN thin film were deposited using PLD, with deposition details included in the Experimental Methods section. To determine the phase and orientation of the phases, XRD was performed, with the results reported in Figure 1C. There are no obvious oxide peaks (e.g., NiO or Fe_2_O_3_) identified in both the single layer and the VAN thin films, suggesting that the vacuum deposition conditions prevent the oxidation of metallic NiFe. All materials grew as the cubic phases, matching well with the cubic STO substrate, without the formation of any secondary material phases. The broad NiFe (*00 L*) peaks can also be compared between the single layer and VAN films, with the peaks of the single‐layer sample being much broader than those of the VAN films, suggesting a better texture of NiFe (*00 L*) in the VAN film. The broader peaks in the single‐layer film could also indicate the presence of microstrain, which also contributes to XRD peak broadening due to internal stress within the film and lattice mismatches with the substrate. A possible factor for the stronger texture of the NiFe in the VAN film is that the cogrowth of the NiFe nanopillars in the CeO_2_ matrix allows the preferential out‐of‐plane (OOP) texture of the NiFe nanopillars. Instead, through the strain compensation model, the growth of NiFe in CeO_2_ becomes more textured.^[^ ^31^ ^]^ It is also noted that the oxide matrix, CeO_2_, did not oxidize the NiFe nanopillars during the vacuum growth. Looking ahead to **Figure** **2**, the TEM data shows the discontinuous island‐like nature of the pure NiFe film with the island sizes ranging from 4.78 to 9.51 nm in height. When embedded in the CeO_2_ matrix in the VAN film, NiFe grows as very straight and thin nanopillars throughout the CeO_2_ matrix. This further suggests that the highly textured growth of NiFe nanopillars is facilitated by the CeO_2_ matrix.

TEM, STEM, and EDS imaging were conducted on the two samples, as shown in Figure 2, to determine the exact microstructure of the films and to better understand the growth of the CeO_2_‐NiFe VAN film. The top half of the figure features the pure NiFe film with a discontinuous island‐like growth as individual NiFe nanoparticles. Figure 2A1 shows a STEM image of the NiFe growth, while Figure 2A2 shows the corresponding EDS mapping for Sr and Ni. Other elements for the NiFe pure film can be found in Figure S1, Supporting Information. The average thickness of these islands measured 7.12 nm. As discussed previously, the large number of islands resulted in peak broadening for the XRD. The enlarged view of one NiFe island, shown in Figure 2C, shows a clear interface between the STO substrate and the NiFe island. NiFe has a smaller lattice parameter (*a* = 3.600 Å) than that of the STO (*a* = 3.91 Å). This large mismatch could result in a domain matching relation that is, 12 of the NiFe lattices match with 11 of STO lattices, leading to misfit dislocations at the interfaces. Figure S3, Supporting Information shows both 10 μm and 500 nm images for the NiFe island and CeO_2_‐NiFe VAN films to demonstrate the spacing of the NiFe islands and the VAN surface roughness. From Figure S3A, Supporting Information, the NiFe islands do remain as discrete entities when viewing the whole plane. The line scan in Figure S3B,D, Supporting Information, shows the particle height of 10–13 nm, which supports the average island thickness seen in the TEM image. This lack of interaction between the NiFe islands could result in lower magnetic properties than if a continuous single layer had been deposited, but due to the VAN pillars being discontinuous from each other, it allows for an interesting comparison. For the CeO_2_‐NiFe sample, Figure S3E–H, Supporting Information show that dust settled on the AFM sample, but otherwise the surface roughness is minimal, less than 5 nm, especially when compared with the single‐layer film. Figure 2D shows a bright field TEM image of the CeO_2_‐NiFe VAN film, with the start of individual pillars marked out with blue arrows. The thickness of this film is estimated to be 107 nm, a 10 time increase in thickness when compared to the single‐phase NiFe film. This supports the idea that the CeO_2_ helped the NiFe grow more vertically. Based on how the NiFe pure film grew as islands, it suggests that the NiFe continued to grow separately into the nanopillars while the CeO_2_ grew as the matrix around it.

Figure 2E shows the selected area electron diffraction (SAED) pattern from the VAN film, with the STO and CeO_2_ diffraction spots labeled. Due to the NiFe pillars being so thin and separated, individual NiFe diffraction spots are relatively weak in the SAED pattern and thus could not be identified. Figure 2F1,F2 show the STEM image and EDS mapping for the CeO_2_‐NiFe VAN film, respectively. In the high‐angle annular dark‐field (HAADF) imaging mode in STEM, individual elements show different contrast based on their atomic number Z values (so‐called Z‐contrast imaging). The NiFe shows a brighter contrast compared to that of the matrix. It is also seen that some of the NiFe pillars appear to terminate early, this could be due to some pillars being shorter or because the wedge‐type TEM foil is much thinner on the edge and some of the pillars could be truncated. To demonstrate how the wedge shape of the TEM foil is able to truncate pillars, a schematic drawing can be seen in Figure S4, Supporting Information demonstrating how different thickness of the wedge can lead to different pillar heights in one cross‐section TEM foil. The average diameter of the NiFe pillars is estimated to be 5.5 nm with the linear density and pillar density determined to be 9.03 × 10^5^ pillars/cm and 8.15 × 10^11^ pillars/cm^2^, respectively, based on the full HAADF and Ni EDS map.

**Figure 3:**
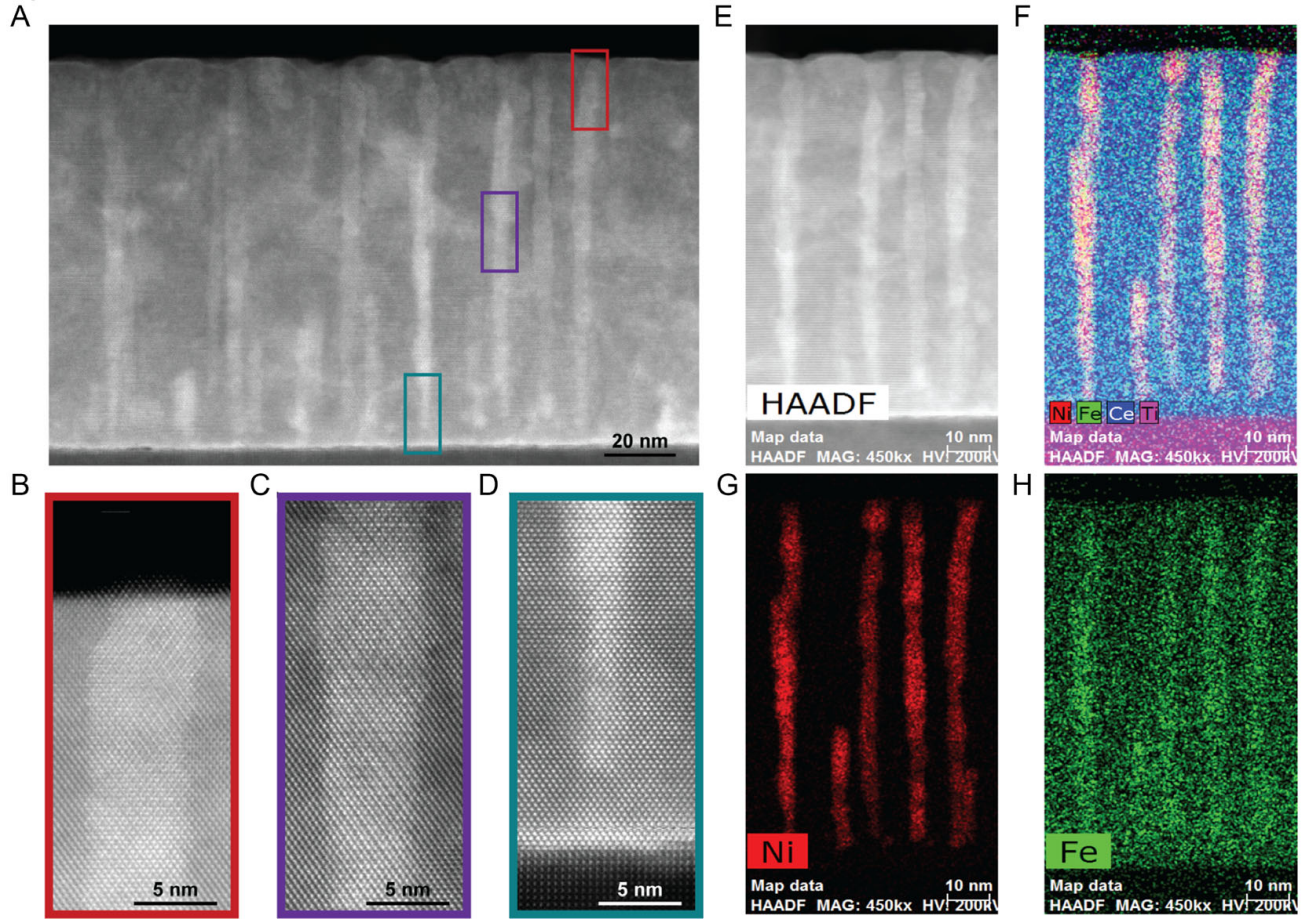
For the CeO_2_‐NiFe VAN system: A) High‐resolution STEM image of the sample with areas marked representing the enlarged views seen in B–D. B) Enlarged view of the top of a NiFe pillar, C) Enlarged view of the middle of a NiFe pillar, D) Enlarged view of the bottom of a NiFe pillar. E) STEM image for the corresponding EDS maps, F) EDS map showing Ni, Fe, Ce, and Ti, G) EDS map showing only Ni, and H) EDS map showing only Fe.

High‐resolution TEM, STEM, and EDS imaging were also performed on the CeO_2_‐NiFe VAN film, allowing for a more detailed view of the film morphology and crystal structures within the film, as seen in **Figure** **3**. Figure 3A shows a large area of the film with three sections marked out representing the top, middle, and bottom of a NiFe pillar. From Figure 3A, it is apparent that when viewing the top of the film, the CeO_2_ grew a few atom layers higher than the NiFe. This supports the idea that the NiFe pillars are still strained and that the CeO_2_ matrix helped the NiFe grow throughout the film. Figure 3B,D shows the enlarged views of the top, middle, and bottom of three different NiFe pillars. In Figure 3B, the top of a NiFe pillar is shown, and it can be seen that the top of the pillar does grow through the whole film without a CeO_2_ layer on top of it. The shape and size of the pillars can vary throughout the thickness, continuing into Figure 3C. To resolve the strain caused by the different lattice spacing of CeO_2_ and NiFe (5.411 and 3.600 Å respectively), the pillar changes its shape. However, the lattice spacing within the pillar remains consistent. This can be seen from how the atomic lattices extend clearly from the matrix to the pillar. Near the substrate, as in Figure 3D, it can be seen that the pillar does not contact the STO substrate. Instead, a few layers of CeO_2_ are grown first because CeO_2_ has a lower surface energy for the initial growth when compared to NiFe. This CeO_2_ layer initially appears brighter than the further layers of CeO_2_ grown in the film, but as can be seen in Figure 3E–H no Ni or Fe occurs significantly in that layer of CeO_2_. Though some energies of Fe and Ce overlap slightly, causing Figure 3H to appear to have Fe in the CeO_2_ matrix. Going back to the initial CeO_2_ layer grown, the bright color in the STEM image is due to Ce being a heavy element and not having another material to contrast against, which happens when compared with the NiFe pillars. The Figure 3G clearly shows that the Ni component in the pillars is well constrained to the shape of the pillars seen in the corresponding image. The ratio of Ni to Fe stays consistent throughout the height of each pillar.

**Figure 4:**
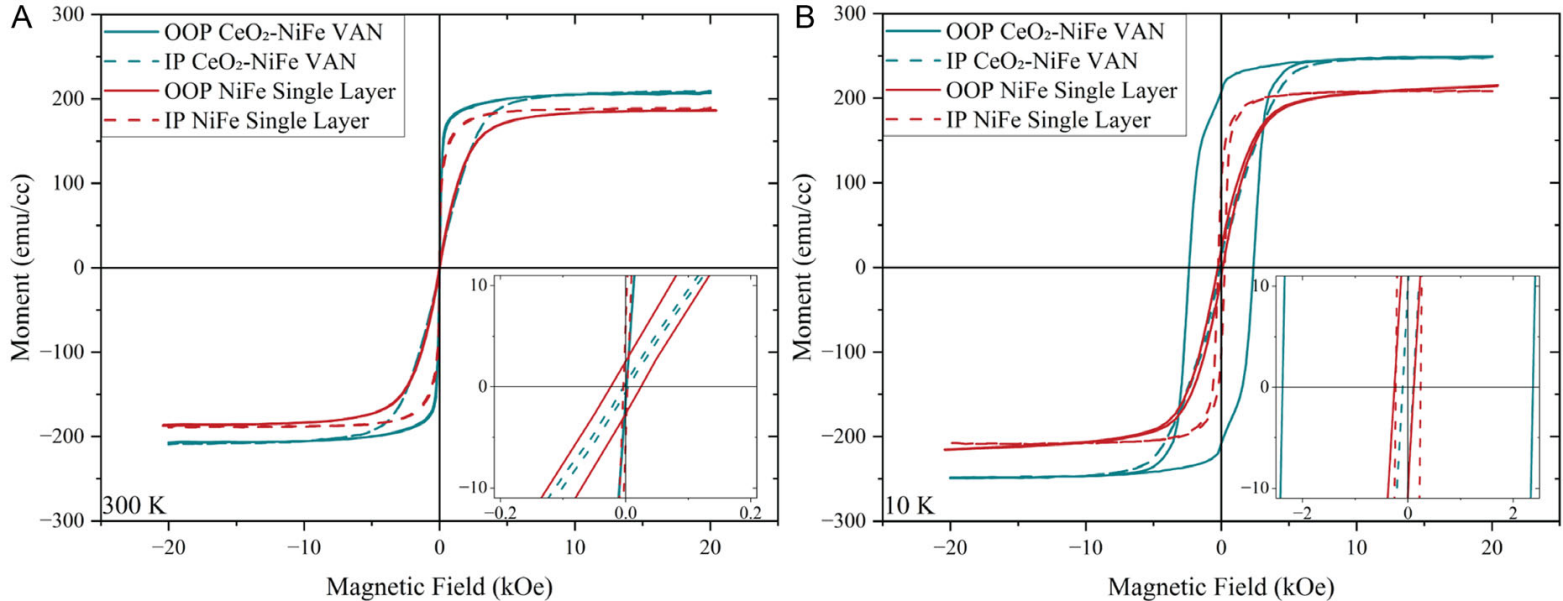
Graphs showing the magnetic moment versus magnetic field at A) 300 K and B) 10 K for both the NiFe single layer and the CeO_2_‐NiFe VANs.

Considering the very different NiFe morphologies, the physical properties could be very different. First, magnetic hysteresis loops were measured, as seen in **Figure** **4** below with 4A for the room temperature (300 K) measurements and 4B for the low temperature (10 K) measurements. For the NiFe pure film with isolated islands and domains, a weaker anisotropy is expected, as there is no obvious shape anisotropy. For the CeO_2_‐NiFe VAN film, the nearly perfect vertical NiFe nanopillars are expected to present strong shape anisotropy, that is, preferred OOP anisotropy. Based on the data seen in Figure 4, it can be confirmed that the VAN sample has a more obvious OOP anisotropy than the NiFe single‐layer sample and such a trend is more obvious for the 10 K measurement. Specifically, the 10 K data suggests an obviously much larger coercive field in the OOP loop of the VAN sample (≈2.4 Oe) than the IP value (≈0.06 Oe). Such shape anisotropy is much less obvious for the NiFe single‐layer sample. It confirms that the shape anisotropy contributed by the highly vertically aligned NiFe nanopillars. Both films present comparable magnetization strength with slightly stronger magnetic moment in the VAN film, suggesting the overall magnetic moment in the two films is comparable. This demonstrates that by growing the NiFe in VAN form, the magnetic shape anisotropy can be effectively tuned from random in the single‐layer case to a strong OOP anisotropy in the VAN film.

**Figure 5:**
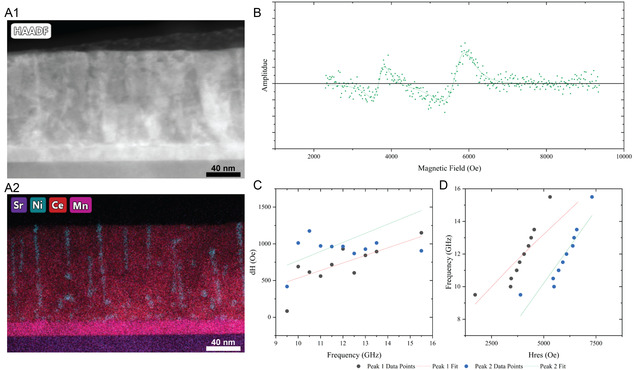
For the CeO_2_‐NiFe sample with the LSMO buffer layer A1) STEM image and A2) corresponding EDS map representing the Sr, Ni, Ce, and Mn elements. B) Shows the 11 GHz data collected during FMR measurements. C) Graph showing the width of the two frequency peaks versus the frequencies they appear at. D) Graph showing the frequency resonance peaks appear at versus where the peaks occur.

Considering the potential magnonic properties of the NiFe nanostructures, FMR measurements were conducted. FMR explores the dynamics of magnetic moments under an applied magnetic field, which is important for spintronics applications. A signature parameter to the measurement is the samples’ Gilbert damping coefficient (α), and NiFe is well‐known to have a low α. It was believed that the new microstructure would produce a α value similar to the measured values from pure single‐layer NiFe or patterned NiFe previously reported, in the range of 1 × 10^−2^ to 1.5 × 10^−3^.^[^ ^15^, ^16^, ^17^, ^18^ ^]^ The VAN film containing CeO_2_ would only have the NiFe pillars contributing to the measured resonance since CeO_2_ is diamagnetic. To allow the conductive spin transport measurement, a thin magnetic buffer layer of La_0.7_Sr_0.3_MnO_3_ (LSMO) was implemented for the VAN film to connect all the discontinuous pillars. The buffer layer was decided to be a different magnetic material so that the resonance peaks of the buffer layer and the NiFe pillars could be measured separately from each other. Specifically, LSMO was first deposited, followed by the growth of the CeO_2_‐NiFe VAN film. The STEM and EDS image of this new film can be seen in **Figure** **5**A1 and A2. Further TEM and magnetic hysteresis loop measurements for the LSMO/CeO_2_‐NiFe film can be found in Figure S6, Supporting Information. When grown on the LSMO buffer layer, the CeO_2_‐NiFe VAN microstructure on the buffer layer is comparable to the one directly grown on the STO substrate with minor deterioration of the pillar nanostructure, likely due to LSMO lattice parameter being slightly larger than STO.

As seen in Figure 5B, FMR was able to capture the response of the CeO_2_‐NiFe VAN sample on LSMO buffer. A set of measurements were conducted as a function of the frequency, measured between 9.5 and 15.5 GHz. Due to each frequency having two resonance peaks from the LSMO and NiFe materials, only one frequency, 11 GHz, is shown here as a representative. For reference, all the other frequencies measured can be found in Figure S7, Supporting Information. Figure 5C shows the width of each response at every measured frequency, and Figure 5D plots the measured frequencies versus the field at which the resonance occurs. The fits of these two graphs allow for α to be calculated along with other parameters. The α for the first peak is 5.29 × 10^−2^ and the α for the second peak is 3.46 × 10^−4^. These two values are in the reported range for NiFe and LSMO films, respectively, showing that FMR could be measured on the CeO_2_‐NiFe VAN film with a thin LSMO buffer layer.^[^ ^13^, ^14^, ^15^, ^16^, ^17^ ^]^

The magnetic damping of NiFe found in this VAN material, 5.29 × 10^−2^, is at the higher end of values typically reported for NiFe. There are a few different possible explanations for why this could have occurred. First, the density of the NiFe nanopillar is relatively low with a pillar density of 8.15 × 10^11^ pillars/cm^2^. A higher density of NiFe pillars could also lead to better magnon dynamic properties. Second could be the inhomogeneity of the VAN film. The nature of magnetic pillars in a diamagnetic matrix forces non‐uniform magnetic fields to form within the film. These variations can act as scattering centers for magnons, promoting processes such as two‐magnon scattering, where the uniform precession mode couples to degenerate spin‐wave modes. This leads to enhanced energy dissipation, leading to FMR peak broadening and increasing the effective magnetic damping.^[^ ^38^ ^]^ Third, the higher strain and anisotropy introduced by the VAN microstructure may cause spatial variation in local resonance conditional along the individual pillars. This non‐uniformity prevents the coherent precession across the entire film, due to the diamagnetic matrix, and further contributes to linewidth broadening and increased magnetic damping.^[^ ^39^ ^]^ Increasing the NiFe pillar density and improving the growth of the VAN on top of the LSMO buffer or a different buffer layer with better lattice matching and could improve the Gilbert damping coefficient in the future. This proof of concept of VAN‐based NiFe nanopillars still works and could be used on other nanostructures with discontinuous magnetic domains.

Based on the above microstructure and magnetic property analysis, it is clear that the new CeO_2_‐NiFe VAN system offers novel structure and property anisotropy based on the unique vertical NiFe nanopillar designs. Previous work has been done on other CeO_2_‐metal VAN systems, including CeO_2_‐Ni, CeO_2_‐Au and CeO_2_‐Co.^[^ ^34^, ^35^, ^36^, ^37^ ^]^ This is the first demonstration of NiFe permalloy nanopillars in the CeO_2_ matrix as a magnetic hybrid metamaterial. Prior work on lithography‐patterned NiFe nanostructures such as nanowires and nanotubes present a Gilbert damping coefficient of 1 × 10^−2^ to 1.5 × 10^−3^, with mostly in‐plane magnetic anisotropy.^[^ ^15^, ^16^, ^17^, ^18^, ^28^ ^]^ Differently, in this work, because of the vertical pillars, the CeO_2_‐NiFe VAN system shows strong OP anisotropy and a Gilbert damping coefficient of 5.29 × 10^−2^. This self‐assembled NiFe VAN system also presents great potential in achieving tunable magnetic and magnonic transport properties, such as varying the nanopillar diameters by varying the deposition parameters and density of the pillars by tailoring the NiFe composition in the film. Future work with the CeO_2_‐NiFe system could implement a higher NiFe pillar density, becoming more like that seen in the CeO_2_‐Ni system and determining how this affects the properties.^[^ ^35^ ^]^ A higher density of NiFe pillars may have more consistent magnon dynamics. In addition, the matrix materials can be varied to tune the lattice strain in the matrix. Other oxides of interest include ferroelectric BaTiO_3_ and piezoelectric ZnO, which have previously been demonstrated in VAN systems with ferromagnetic materials like BaTiO_3_‐Fe and ZnO‐Ni and ZnO‐Co.^[^ ^40^, ^41^, ^42^, ^43^ ^]^ The versatile selections of the matrix materials add additional tailorability in the NiFe VAN designs toward well‐controlled magnon transport properties via external stimuli such as forces and bias. In addition, recent flexible VANs by transfer also add in the possibility of being freestanding VANs as another future direction that could enable nanostructured spintronics integrated as flexible electronics.^[^ ^44^, ^45^, ^46^ ^]^

## 3 Conclusion

In this work, NiFe (80:20) nanopillars were incorporated in the CeO_2_ matrix as a NiFe‐based VAN thin film to show the effects of nanocomposite designs on the magnetic properties of NiFe when compared to single‐layer NiFe. The VAN films showed better NiFe growth than that of the single‐layer sample when comparing the microstructure, suggesting that the VAN microstructure and CeO_2_ helped with the growth of NiFe. The CeO_2_‐NiFe VAN films show a strong OP magnetic anisotropy and a higher overall magnetic saturation than the NiFe single‐layer film. FMR measurements show that the self‐assembled NiFe‐CeO_2_ hybrid metamaterial with LSMO buffer layer presents a Gilbert damping coefficient of 5.29 × 10^−2^. Future work could focus on VAN films with different NiFe pillar density and pillar diameters and different matrix materials for tunable magnon transport properties. This study shows that the one‐step self‐assembled VAN structures present great potential for future spintronics and magnonic devices with NiFe or other nanostructures as the conducting films.

## 4 Experimental Methods

The CeO_2_ target was prepared by pressing pure CeO_2_ powder into a target and sintering for 10 h at 1,200 °C. The Ni_80_Fe_20_ strip and plate were purchased from Goodfellow, with the strip being pasted to the CeO_2_ target during deposition with silver paste. The strip was cut from the plate with a slow‐speed saw and had a width of 3.5 mm. The La_0.7_Sr_0.3_MnO_3_ (LSMO) target was made by mixing La_2_O_3_, SrO_3_, and MnO_2_ powders stoichiometrically to form the 0.7:0.3:1 ratio. It was then sintered for 10 h at 1200 °C.

All the thin film samples were made with PLD with a KrF excimer laser (Lambda Physik, *λ* = 248 nm) with a laser energy of 420 mJ. For the NiFe single‐layer film a vacuum background pressure and a pulse number of 8,000 was used. The CeO_2_‐NiFe VAN film used a vacuum background pressure and 3,000 pulses. The thin film with the LSMO buffer layer deposited the LSMO layer with a background pressure of 150 mTorr O_2_ and 1,000 pulses. The chamber was then reduced back to vacuum, and the CeO_2_‐NiFe VAN layer was then deposited in the same method as in the film without a LSMO buffer. All three samples were deposited at 800 °C with a laser frequency of 5 Hz on commercial STO (100) substrates. After deposition for all samples, they were cooled to room temperature at a rate of 15 °C min^−1^ under a vacuum background pressure. The samples were then cut into roughly 5 × 5 mm wide pieces.

Crystallinity, elemental, and microstructural characterization were performed with a PANalytical Empyrean XRD with a Cu Kα radiation source, high‐resolution Thermo Fisher Scientific TALOS 200 × STEM operated at 200 kV, and a FEI Titan G2 80–200 STEM with a Cs probe corrector and ChemiSTEM technology (X‐FEG and SuperX energy‐dispersive X‐ray spectroscopy with four windowless silicon drift detectors) also operated at 200 kV. Sample preparation was done by thinning the sample through manual grinding and polishing followed by dimpling and more polishing. The sample was then finished with a PIPS II Model 695 ion miller from Gatan.

Magnetic hysteresis measurements were completed with an MPMS Model 3 (Quantum Design) with EverCool SQUID magnetometer in the user facility of the Birck Nanotechnology Center at Purdue University see birck.research.purdue.edu. The magnetic moment versus applied field measurement was completed in both the in‐plane and OOP direction at both 300 and 10 K to a field of 20 kOe. The FMR measurements were completed with a PPMS DynaCool (Quantum Design) and a CryoFMR add‐on (NanOsc) in the user facility of the Birck Nanotechnology Center at Purdue University, see birck.research.purdue.edu. The frequency range of the microwave source used was 2–18 GHz, measured at each whole number. The sample was placed on a coplanar waveguide in the in‐plane direction, or with the applied magnetic field being along the length of the film, and the measurement was performed at 300 K. The instrument measured the variation of the absorbed power as a function of the magnetic field (dP/dH) at the mentioned frequencies. The resonance field (*H*_res_) and peak‐to‐peak linewidth (Δ*H*) of the FMR graphs were measured at each frequency and for each peak that occurred with the NanOsc program. The program was then able to calculate other reported parameters such as the Gilbert damping coefficient (α) and the effective saturation magnetization (4πM_eff_) based on the usual equations.

## Conflict of Interest

The authors declare no conflict of interest.

## Supporting information

Supplementary Material

## Data Availability

The data that support the findings of this study are available in the supplementary material of this article.
