# Supplementary material for: Strong Structural and Property Anisotropy in CeO2‐NiFe Hybrid Metamaterials Toward Self‐Assembled Magnon Nanostructures
Source: Small Sci. 2025 Aug 6;5(11):2500070. doi: 10.1002/smsc.202500070 (PMC12622530; doi:10.1002/smsc.202500070)
Supplement: Supplementary file 1 — Supplementary Material [file SMSC-5-2500070-s001.pdf]

Supplementary Information

Strong Structural and Property Anisotropy in CeO_2-NiFe Hybrid Metamaterials towards Self-Assembled Magnon Nanostructures

Lizabeth Quigley,[1] Juanjuan Lu,[1] Claire A. Mihalko,[1] Jialong Huang,[1] Jeremy Gan,[1] Katrina Evancho,[1] Max Chhabra,[1] Raktim Sarma,[2, 3] Aleem Siddiqui,[2 ,3] Ping Lu,[2] Haiyan Wang[1, 4*]

[1] School of Materials Engineering, Purdue University, West Lafayette, Indiana 47907, USA

[2] Sandia National Laboratory, Albuquerque, New Mexico, USA

[3] Center for Integrated Nanotechnologies, Sandia National Laboratory, Albuquerque, New Mexico, USA

[4] School of Electrical and Computer Engineering, Purdue University, West Lafayette, Indiana 47907, USA

*Author to whom correspondence should be addressed. E-mail: hwang00@purdue.edu

The supporting information includes the following information.

Figure S1: For the pure NiFe film: A) STEM image, the rest are corresponding EDS images for B) O element, C) Ni element, D) Fe element, E) Sr element, F) Ti element, and G) Sr and Ni elements.

Figure S2: For the CeO_2-NiFe VAN film: A) STEM image, the rest are corresponding EDS images for B) O element, C) Ni element, D) Fe element, E) Sr element, F) Ti element, G) Sr, Ni, and Ce elements, and H) Ce element.

Figure S3: AFM data for the NiFe single layer film: A) 2D 10 µm image, B) 2D 500 nm image, C) 3D 500 nm image and D) film roughness graph from the line indicated in B). AFM data for the CeO_2-NiFe VAN film: E) 2D 10 µm image, F) 2D 500 nm image, G) 3D 500 nm image and H) film roughness graph from the line indicated in F).

Figure S4: Schematic drawing illustrating the A) Wedge shape of a TEM sample and the ability to truncate pillars based on their position in the TEM foil and B) The view of the complete and truncated pillars when viewing the TEM sample straight on.

Figure S5: For the $La_{0.7}Sr_{0.3}MnO_3$ (LSMO) buffer layer sample: A) STEM image, the rest are corresponding EDS images for B) O element, C) Ni element, D) Fe element, E) Sr element, F) Ti element, G) Mn element, H) Sr, Ni, Ce, and Mn elements, and I) Ce element. J) Large scale TEM image. K) Enlarged TEM image. L) Larger scale STEM image.

Figure S6: For the $La_{0.7}Sr_{0.3}MnO_3$ (LSMO) buffer layer sample: A) XRD θ-2θ scan, magnetic moment versus magnetic field at B) 300 K and C) 10 K.

Figure S7: For the For the $La_{0.7}Sr_{0.3}MnO_3$ (LSMO) buffer layer sample: A) All frequencies collected during FMR measurements, B) The width of the two peaks versus the frequencies they appear at. D) The frequency peaks appear at versus where the peaks occur.

Figure S8: XRD graphs representing the different CeO2-NiFe VAN deposition attempted, with differences in A) energy (temperature consistent at 800°C) and B) temperature (energy consistent at 420 mJ) to determine the best deposition conditions.

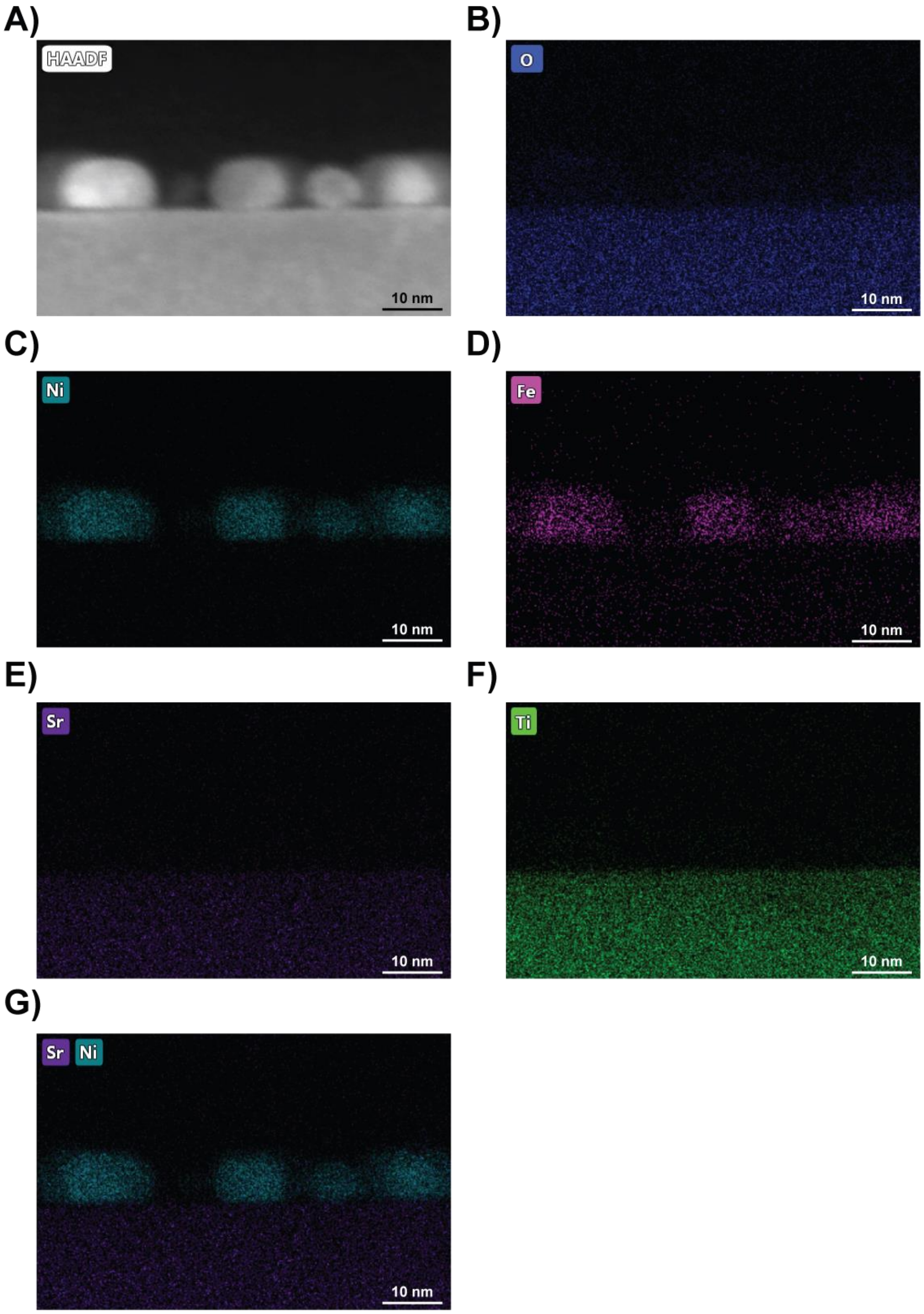

Figure S1: For the pure NiFe film: A) STEM image, the rest are corresponding EDS maps for B) O element, C) Ni element, D) Fe element, E) Sr element, F) Ti element, and G) Sr and Ni elements.

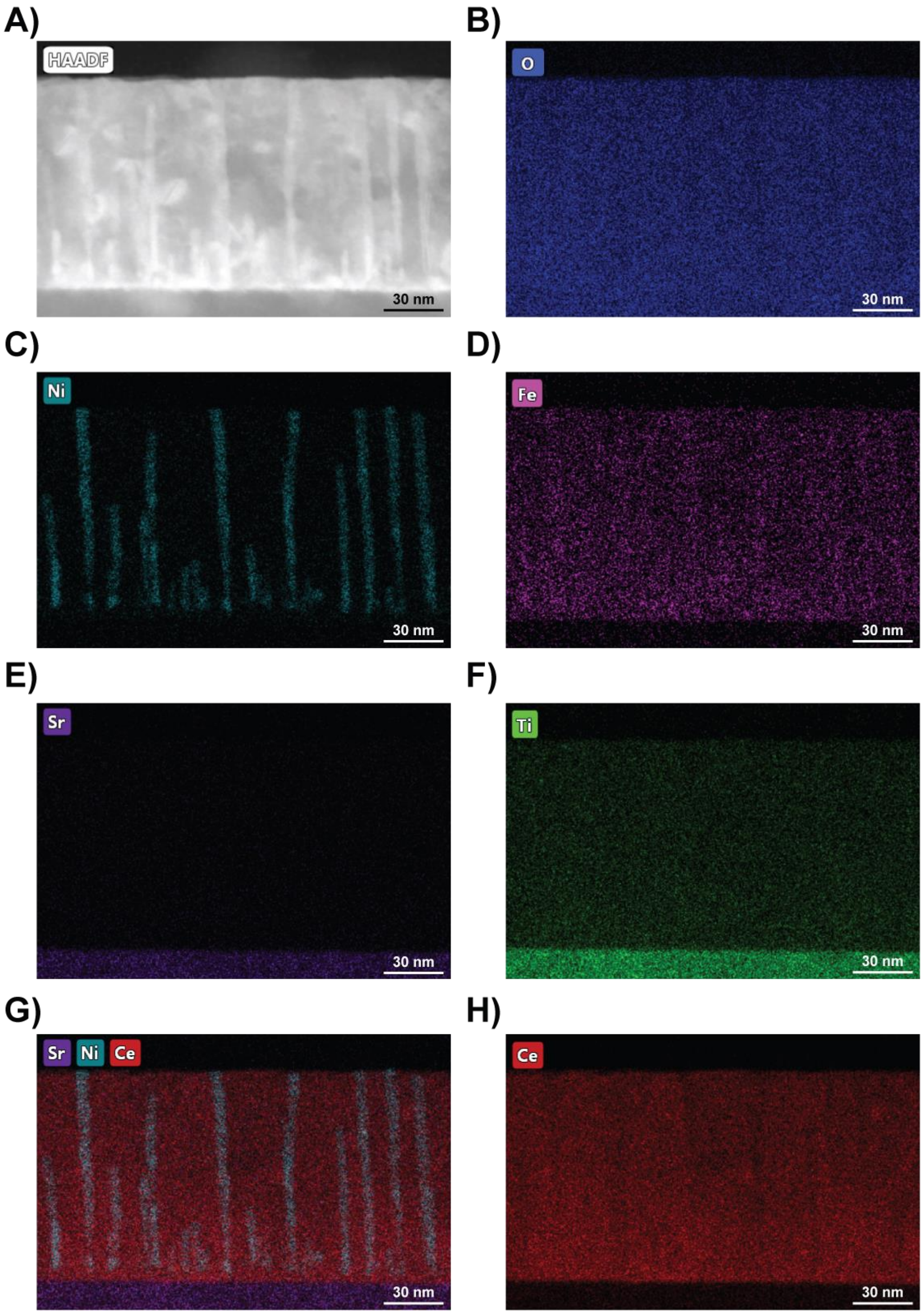

Figure S2: For the CeO_2-NiFe VAN film: A) STEM image, the rest are corresponding EDS maps for B) O element, C) Ni element, D) Fe element, E) Sr element, F) Ti element, G) Sr, Ni, and Ce elements, and H) Ce element.

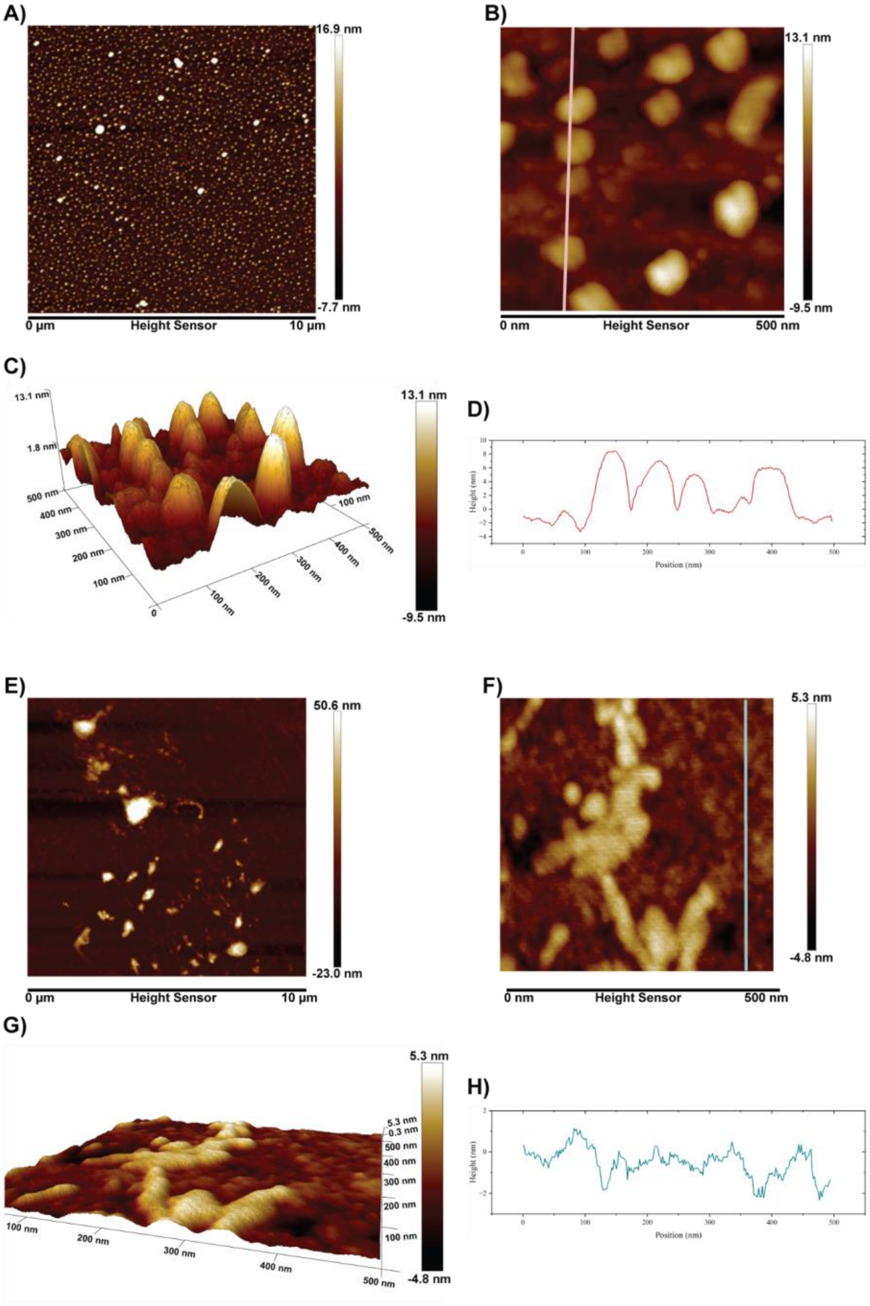

Figure S3: AFM data for the NiFe single layer film: A) 2D 10 µm image, B) 2D 500 nm image, C) 3D 500 nm image and D) film roughness graph from the line indicated in B). AFM data for the CeO_2-NiFe VAN film: E) 2D 10 µm image, F) 2D 500 nm image, G) 3D 500 nm image and H) film roughness graph from the line indicated in F).

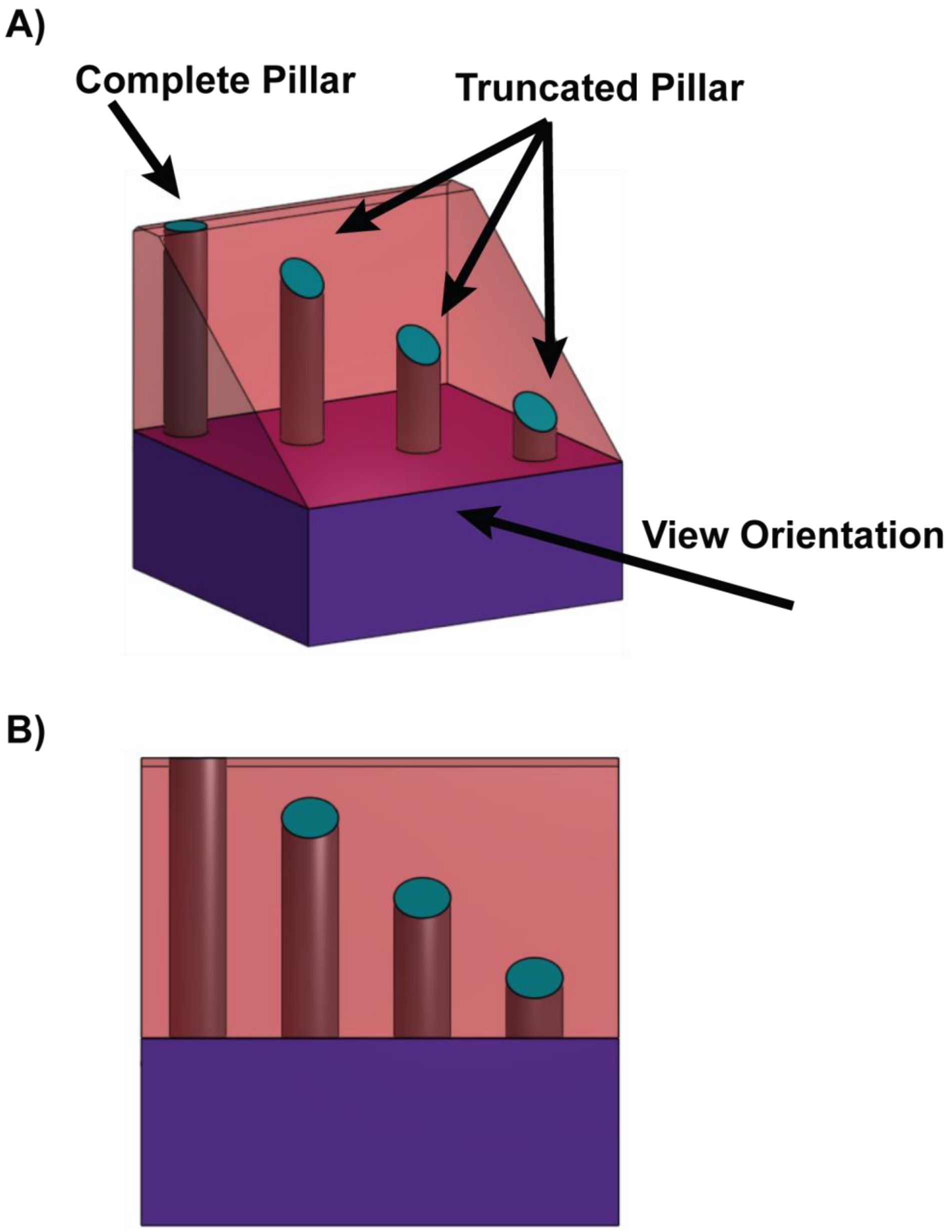

Figure S4: Schematic drawing illustrating the A) Wedge shape of a TEM sample and the ability to truncate pillars based on their position in the TEM foil and B) The view of the complete and truncated pillars when viewing the TEM sample straight on.

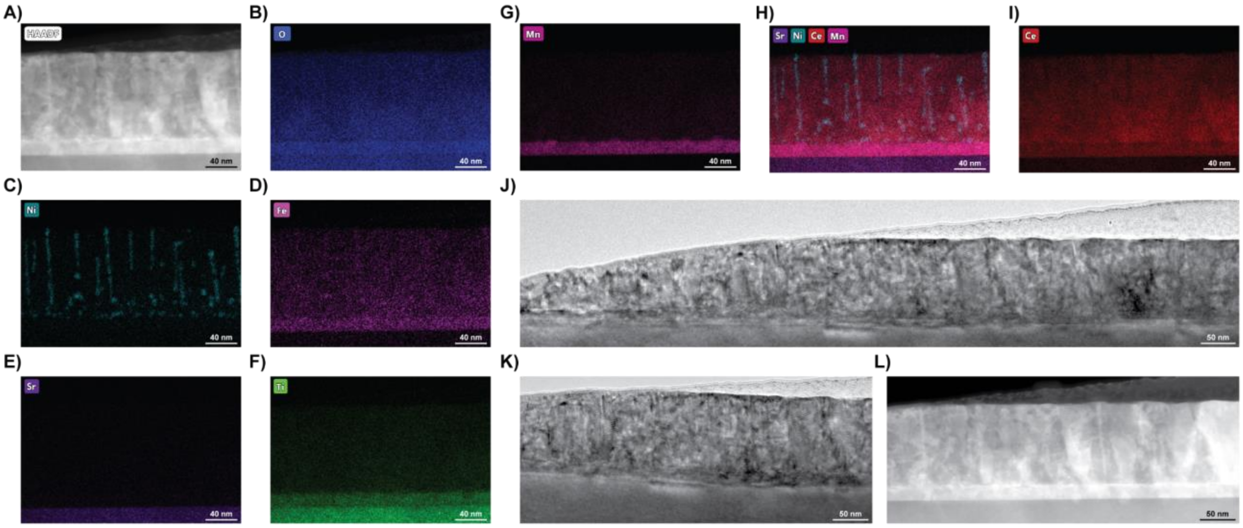

Figure S5: For the $La_{0.7}Sr_{0.3}MnO_3$ (LSMO) buffer layer sample: A) STEM image, the rest are corresponding EDS maps for B) O element, C) Ni element, D) Fe element, E) Sr element, F) Ti element, G) Mn element, H) Sr, Ni, Ce, and Mn elements, and I) Ce element. J) Low-magnification TEM image. K) Higher-magnification TEM image. L) Larger range STEM image.

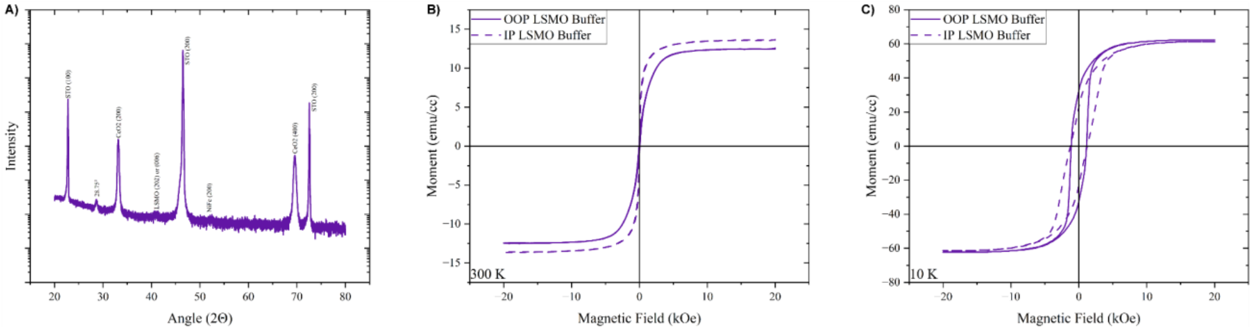

Figure S6: For the $La_{0.7}Sr_{0.3}MnO_3$ (LSMO) buffer layer sample: A) XRD θ-2θ scan, Magnetic moment versus magnetic field at B) 300 K and C) 10 K.

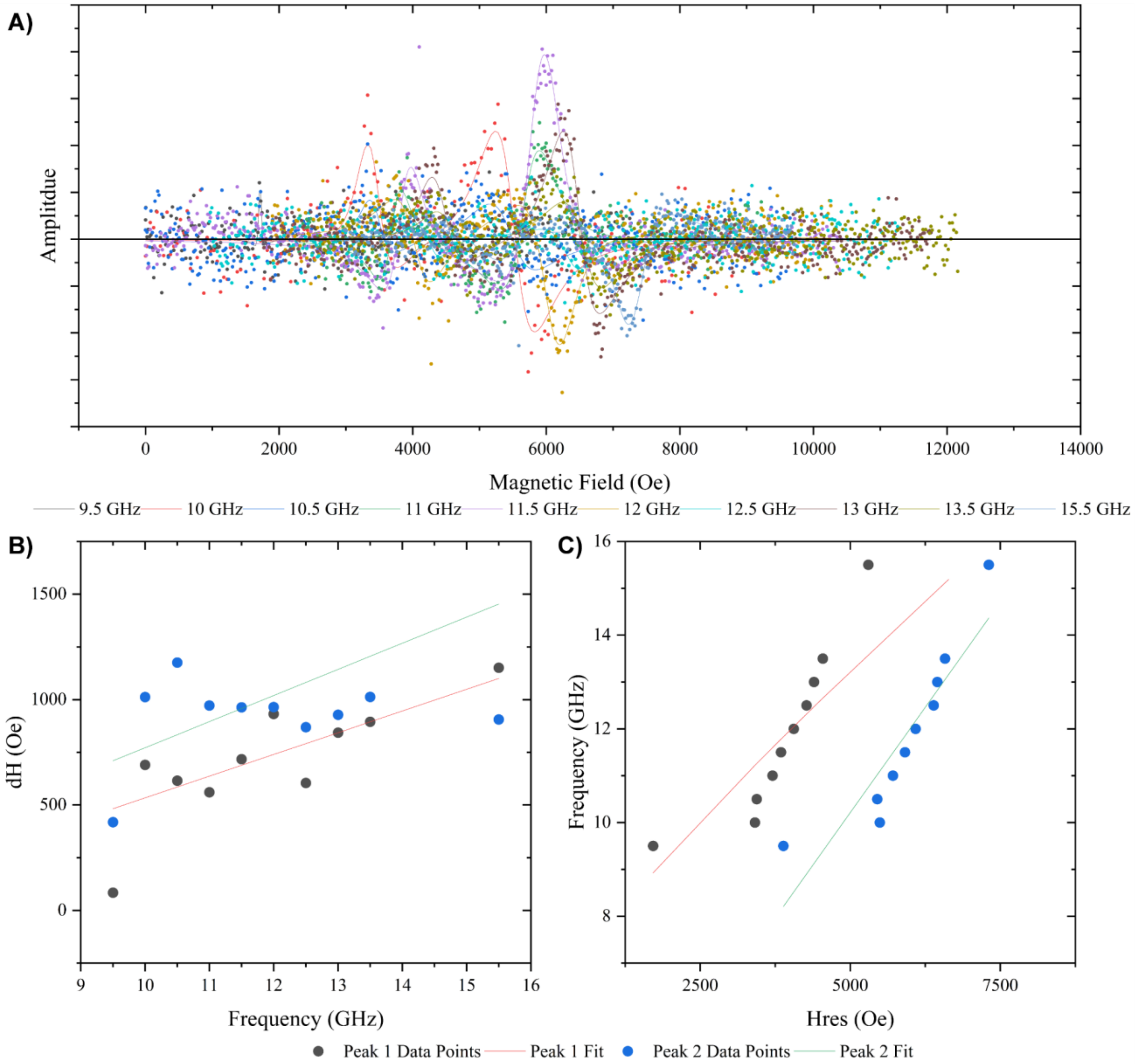

Figure S7: For the For the $La_{0.7}Sr_{0.3}MnO_3$ (LSMO) buffer layer sample: A) All frequencies collected during FMR measurements, B) The width of the two peaks versus the frequencies they appear at. C) The frequency peaks appear at versus where the peaks occur.

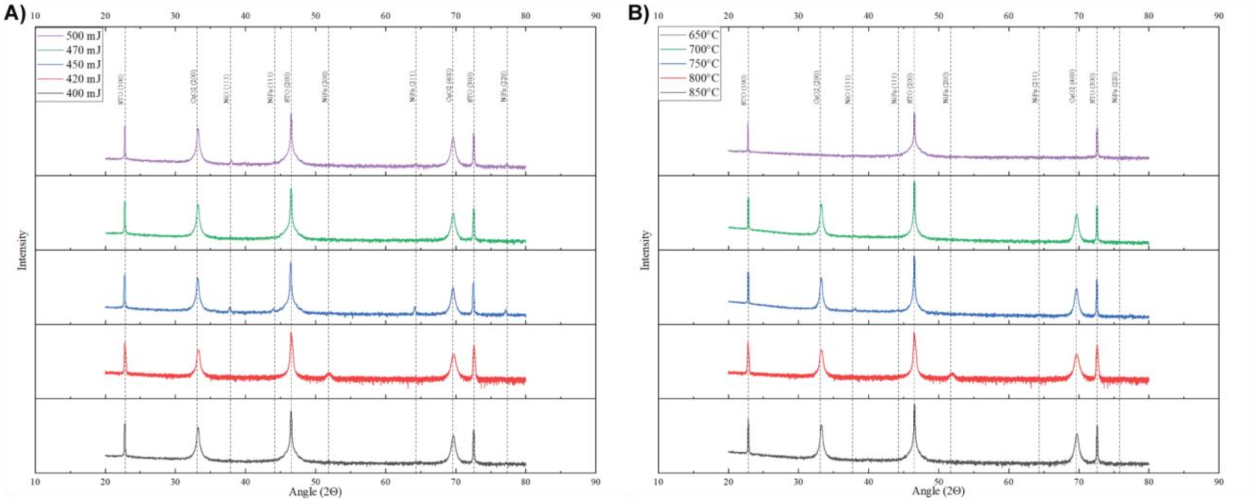

Figure S8: XRD graphs representing the different CeO2-NiFe VAN deposition attempted, with differences in A) energy (temperature consistent at 800°C) and B) temperature (energy consistent at 420 mJ) to determine the best deposition conditions.
